# Medical News and Miscellany

**Published:** 1888-06

**Authors:** 


### MEDICAL NEWS AND MISCELLANY.

DR. Y. D. HARRINGTON, who left Terrell, Texas, for Los Angeles, California, a year or so ago, has returned to Terrell.

DR. C. K. GREGG, son of the venerable Bishop Gregg, of Texas, (Episcopal), has returned from Mexico, where he has resided for several years and has settled in McKinney, Collin county.

DR. R. P. TYE, whose removal we mentioned last month, has moved again; this time to Clarendon.

DR. J. M. ROSS, formerly of Brenham, removed to Belton, thence to Dallas. He has returned to his first love.

DR. B. E. HADRA, of Austin, having accepted the Chair of Surgery in the Texas Medical College, has removed to Galveston.

WE ARE PLEASED to meet in Austin, Dr. Church, late of Calvert, —a fresh graduate of the Baltimore Medical College.

MARRIAGES. Dr. C. M. Rosser, of Waxahachie, was married May 15, to Miss Eva Belknap, of that city, and Dr. L. M. Barnes, of Grandview, to Miss Winburne, in Hill county, May 19, also Dr. Walker to Miss Welsh, in Paris, Texas, May 11.

PHYSICIANS' MUTUAL BENEFIT ASSOCIATION OF TEXAS.—As some of the members held back paying assessment No. 6, waiting to see what, if any, changes would be made at Galveston, notice is hereby given that the amounts stand as before, and those who have not paid, are now called on to do so.

GEO. P. ROWELL, the Newspaper Advertising Directory man, writes us: "It is a fact that less than one paper in sixteen has furnished such a straightout statement of actual issues as you have done."

IS THE WORLD SATISFIED WITH THE MEDICAL PROFESSION?—Dr. L. W. Cock, of St. Marcos, asks this question in his paper in this issue, and answers it in the negative, pointing out the reasons why, and discussing the same in a style that will cure the blues or make a sick man smile. It is "truth in a nutshell," and is replete with humor and satire. Read it and enjoy a smile.

An excellent and valuable paper, by Prof. Rogers, of Memphis, intended for this number, is necssarily omitted on account of the index of the volume. We regret it exceedingly; but it will keep, and our readers will enjoy it the more for the anticipation, we hope.

---

JNO. SOUTHGATE, of Austin, OUR binder, will bind your journals in good style for $1.00 per volume. This volume (Vol. 3) DANIEL'S TEXAS MEDICAL JUORNAL, contains four portraits of eminent Texas physicians, living and dead, and is worth preserving. They are as follows: Dr. A. R. Kilpatrick, Dr. T. D. Wooten, Dr. S. F. Starley and Dr. J. F. Y. Paine. Vol. 2 contains portraits of Drs. W. J. Burt, S. R. Burroughs and R. Rutherford.

